# Recycling Vanadium and Proton-Exchange Membranes from Waste Vanadium Flow Batteries through Ion Exchange and Recast Methods

**DOI:** 10.3390/ma15113749

**Published:** 2022-05-24

**Authors:** Wei-Sheng Chen, Yu-An Chen, Cheng-Han Lee, Yen-Jung Chen

**Affiliations:** Department of Resources Engineering, National Cheng Kung University, No. 1, Daxue Rd., East Dist., Tainan City 701401, Taiwan; kenchen@mail.ncku.edu.tw (W.-S.C.); n46084167@ncku.edu.tw (Y.-J.C.)

**Keywords:** vanadium flow battery, proton-exchange membrane, vanadium, ion exchange, Dowex G26, recovery, recast

## Abstract

This study aims to provide a system to recycle vanadium resources and recover membranes from waste proton-exchange membranes. This research is divided into two parts. To begin, ion exchange batch and column experiments were applied to adsorb vanadium in a membrane. In this process, the waste membrane was initially dissolved in a 50% ethanol solution, and the suspension obtained by dispersing the membrane had 74 mg/L of vanadium. Then, Dowex G26 resin was used to adsorb vanadium from the membrane dispersion in the ion-exchange process. The adsorptive behavior and optimal parameters were explored in this study. The vanadium ions were then eluted by HCl to obtain an enrichment solution, and the V_2_O_5_ was received through precipitation and calcination methods. After obtaining the vanadium-free dispersion, the recycled membrane was prepared by recasting it in the second part. The characteristics of the recycled membrane, such as the moisture, FTIR spectra, ion-exchange capacity, and ion conductivity, are discussed. The results revealed that the adsorption capacity of vanadium through Dowex G26 was 81.86 mg/g. The eluting efficiency of HCl was 97.5%, and the optimal parameters of the precipitation and calcination processes were set as pH 5, NH_4_Cl:V = 2:1, and 350 °C. The moisture of the recycled membrane was 25.98%, and the IEC was 0.565 meq/g. The consequences of FTIR and ion conductivity demonstrated that the vanadium in the recycled membrane was eliminated by the ion-exchange method; however, the microstructure of the recycled membrane was influenced during ion exchange and recasting.

## 1. Introduction

**Figure 1:**
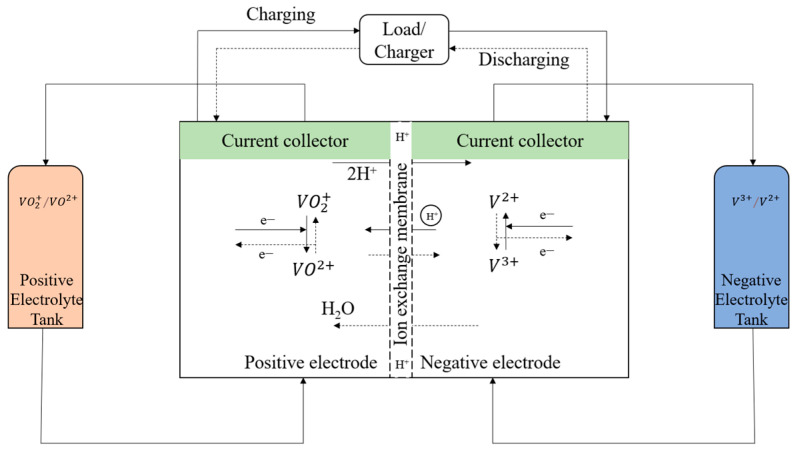
Model of a vanadium redox battery.

The vanadium flow battery (VFB) has attracted considerable attention as a future energy storage system that can offer a megawatt/h storage of the electric energy from renewable energies, including solar energy and wind energy [1,2,3]. According to the report of the U.S. Department of Energy, there are 66 vanadium flow battery energy storage systems in the world [4]. The VFB consists of a stack and two electrolyte tanks. The positive (VO_2_^+^/VO^2+^) and negative (V^3+^/V^2+^) electrolytes are stored in the tank, respectively, and can be pumped into the stack to drive the redox reaction [5] (Figure 1). The positive and negative electrolytes operate between VO_2_^+^/VO^2+^ and V^3+^/V^2+^ in the strong H_2_SO_4_ aqueous solution during the charge and discharge cycle. The electrochemical reactions of positive and negative equations are shown in Equations (1) and (2) [6,7].

Positive: VO_2_^+^ + 2H^+^ + e^−^ ⇌ VO^2+^ + H_2_O (1)

Negative: V^3+^ + e^−^ ⇌ V^2+^ (2)

In the stack, electrolytes are separated by a proton-exchange membrane that makes the proton maintain the electrical balance during the charge and discharge cycle. There are various types of proton-exchange membranes, and the most common is Nafion, which is the primary type discussed in this study. Nafion is a polymer material perfluorinated membrane with strong acid resistance and high proton conductivity [8]. The proton can transfer from side to side through the hydrophilic ion cluster [9]. The high proton conductivity gives the Nafion a great advantage as a VFB proton-exchange membrane but leads to a crucial problem, vanadium fouling.

**Figure 2:**
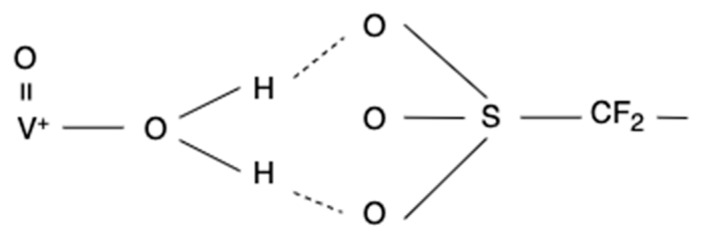
The mechanism of the reaction between vanadium and sulfonic groups.

The literature reported that the proton conductivity will be reduced with continuous VO^2+^ ion penetration [10]. The VO^2+^ ion is observed to bond with the sulfonic acid sites through water protons in the vanadyl ion hydration shell [11]. The mechanism of the reaction between vanadium and sulfonic groups is presented in Figure 2. The sulfonic ion dominated by the vanadium ion was unable to transport protons. The continuous decrease of conductivity will deteriorate the battery capacity and finally make the membrane dysfunctional [12]. This scenario will cause considerable costs and vanadium losses when replacing new membranes during the daily operation of VFB. Therefore, it is necessary to recover the waste membrane and recycle the vanadium resources in the membrane to reach the goal of resource circulation.

There is no research yet reporting the method of recovering the waste membrane from VFB; however, experiments on recovering the proton-exchange membrane of fuel cell batteries have been conducted, and their information is demonstrated in Table 1 [13,14,15,16]. This study aims to provide a simple system to recycle vanadium resources and recover membranes from waste Nafion.

In order to separate vanadium from the waste membrane solution without changing the composition of the membrane ionomer, an ion-exchange method was used in this study to recover vanadium ion from the solution. The ion-exchange process is a reaction conducted by the ion-exchange resin where functional groups can absorb cations and anions from the solution. Through the adsorption and eluting steps, the target ion can be separated. The reaction of ion exchange is shown in Equations (3) and (4). The different techniques of adsorbing vanadium through resins are revealed in Table 2 [17,18,19,20,21].

(3) $$M_{1}^{+}+\mathrm{Rc}\cdot M_{2}\to M_{2}^{+}+\mathrm{Rc}\cdot M_{1}\;(\mathrm{for}\;a\;\mathrm{cation}\text{-}\mathrm{exchange}\;\mathrm{resin})\;$$

(4) $$M_{3}^{-}+\mathrm{Ra}\cdot M_{4}\to M_{4}^{-}+\mathrm{Ra}\cdot M_{3}\;(\mathrm{for}\;\mathrm{an}\;\mathrm{anion}\text{-}\mathrm{exchange}\;\mathrm{resin})\;$$

where $M_{1}^{+}$ and $M_{2}^{+}$ are two different cations; $M_{3}^{-}$ and $M_{4}^{-}$ are two different anions; and Rc and Re are the cation and anion-exchange resin, respectively.

In this study, the vanadium ion was released from the waste membrane by dissolving the membrane in a 50% alcohol solution. The vanadium was formed as VO^2+^ in the waste membrane solution. Therefore, this study used a strong acid cation-exchange resin, Dowex G26, which had high vanadium adsorption capacity, to absorb vanadium. The study of the adsorptive behavior and optimal parameters were explored in the batch and column experiments.

Then, VO^2+^ was eluted by HCl to obtain an enrichment solution, and the V_2_O_5_ was obtained through precipitation and calcination methods. The recycled membrane was prepared after the ion-exchange process by recasting vanadium-free membrane dispersion. The characteristics of the recycled membrane, such as the moisture, FTIR spectra, ion-exchange capacity, and ion conductivity, are discussed. The distinctions between the recycled membrane, commercial membrane, and waste membrane are investigated as well. According to the results, we investigate whether the ion-exchange method can recover vanadium without affecting the composition of the membrane solution.

## 2. Materials and Methods

### 2.1. Materials

A waste Nafion 117 membrane, which underwent a VFB lifespan test, was used as the material for this research. The membrane was dissolved in a 50% ethanol (C_2_H_5_OH, >99.5%, ECHO Chemical, Miaoli, Taiwan) solution to obtain a membrane dispersion where the concentration of vanadium detected by ICP-OES was 74 mg/L (2.96 mg/g in the solid membrane). The detailed characterization information of membrane dispersion is shown in Table 3.

Oxovanadium sulfate (VOSO_4_, 99.9%, Alfa Aesar Haverhill, MA, USA) and a commercial 5% Nafion dispersion were used to make the stimulated waste membrane dispersion for the ion exchange experiment. Other chemicals, such as Dowex G26 resin (Lenntech, Delfgauw, Netherlands), hydrochloric acid (HCl, ≥36.5%, Sigma-Aldrich, St. Louis, MO, USA), ammonium hydroxide (NH_4_OH, 30–33%, Sigma-Aldrich, St. Louis, MO, USA), ammonium chloride (NH_4_Cl, 99.5%, SHOWA, Osaka, Japan), and dimethyl sulfoxide (DMSO, 99%, Sigma-Aldrich, St. Louis, MO, USA), were utilized without further purification.

### 2.2. Equipment

Inductively coupled plasma optical emission spectrometry (ICP-OES 2100DV, PerkinElmer optima 2100 DV; Varian, Vista-MPX, PerkinElmer, Waltham, MA, USA) was applied to detect the concentration of vanadium in the solution. X-ray diffraction (XRD; DX-2700, CEPHAS, Taipei City, Taiwan) was used to analyze the structure of crystalline material. The chemical bonds of the materials were revealed by Fourier Transformation Infrared Spectrometer (FTIR; EQUINOX 55 Bruker-AXS, BRUKER, Billerica, MA, USA). The TG analysis was used to detect the calcination temperature of vanadium product by Thermogravimetry/Differential Thermal Analysis Thermoanalyzer (TG-DTA, NETZSCH-409PC, Netzsh, Selb, Germany). The electrochemical impedance spectroscopy was operated by Solartron Analytical-SI 1260 (SI 1260, Solartron Analytical, Shanghai, China).

### 2.3. Metal Separation and Purification

In this study, the vanadium ion was released from the waste membrane by dissolving the membrane in a 50% alcohol solution. Then, the ion exchange batch and column experiments were conducted using Dowex G26 resin. Dowex G26 is a strong acid exchange resin that can adsorb cations efficiently [22,23]. The adsorptive behavior of vanadium through resin and the optimal parameters of the flow rates and bed volumes were investigated in this research. After that, the vanadium was eluted by HCl to obtain an eluting solution. Finally, the vanadium product was received by ammonium salt precipitation and calcination methods to recover the V_2_O_5_.

#### 2.3.1. Ion Exchange Batch Experiment

The pH value of the membrane dispersion was 2, which made the vanadium ion in the dispersion convert to cation complexion. Dowex G26 was then used to adsorb vanadium from the membrane solution in this research. In the adsorption step, 0.1 g resin was added into the six solutions with the different initial concentrations of VO^2+^ (10, 20, 50, 100, 200, and 400 ppm) and adsorbed VO^2+^ for 24 h. The adsorption isotherms described by means of the Langmuir and Freundlich isotherms were used to investigate the adsorptive behavior of vanadium. In the eluting step, HCl was used to desorb vanadium from resin. The parameters of the eluent molarities (0.1–2 M) in the eluting experiment were set up.

#### 2.3.2. Ion Exchange Column Experiment

In the ion exchange column experiment, the Dowex G26 resin was filled in a column of 4 mL with a diameter of 1.12 cm. The membrane dispersion was pumped into the column with a specific flow rate. The ion exchange was operated through the contact of resin and liquid in the column. The tail liquid was collected by the automatic collector, and the concentrations of vanadium were analyzed by ICP-OES. The breakthrough curve was plotted by the results of the experiment. The breakpoint was defined when the concentration was equal to 5% *C_e_* (concentration of adsorbate in the liquid when the adsorption is in equilibrium). In the eluting part, HCl was an eluting solution to obtain vanadium. In the column experiment, the parameters of the flow rate (0.7, 1, and 1.4 mL/min) and total bed volume (170 BV) were set to obtain the optimal adsorption efficiency.

#### 2.3.3. Precipitation and Calcination

The eluting solution was the solution with a high vanadium concentration obtained after the ion-exchange process. The vanadium ion could be precipitated by controlling the pH value and adding ammonium chloride to gain ammonium metavanadate (NH_4_VO_3_). In the precipitation step, the parameters of pH value (2–7) and nNH_4_Cl:nV (1–5:1) were set to find the optimal precipitation rate. After the precipitation procedure, the vanadium oxide (V_2_O_5_) could be received through calcination, and the operating temperature was determined by the result of TG/DTA.

### 2.4. Membrane Recast

The vanadium-free membrane dispersion could be received after the ion-exchange process. In this study, the dispersion was recast to obtain the recycled membrane, and the characteristics were analyzed as well.

#### 2.4.1. Preparation of the Recycled Membrane

The dimethyl sulfoxide (DMSO) was a low toxicity organic solvent used to replace the solvent of membrane dispersion to obtain a higher mechanical strength of the membrane [24]. The membrane dispersion was placed on a clean Petri dish and heated by oven at 160 °C for 1 h to obtain a recycled membrane. The recycled membrane was boiled in deionized water (DI water) before peeling from the Petri dish and was then stored in DI water.

#### 2.4.2. Characterizations

The characteristics, including the moisture, FTIR, ion-exchange capacity, and ion conductivity, were established. The results were compared with the waste membrane and commercial membrane, which were made by using the same casting process of preparing the recycled membrane.

##### Membrane Moisture

The membrane moisture was measured through the difference between the dry and wet weight. The dry weight (*W_dry_*) of the membrane was measured after drying the membrane at 50 °C for 24 h in the oven. The wet weight (*W_wet_*) was measured after immersing the dried membrane in DI water for 24 h. The membrane moisture was calculated by Equation (5).

(5) $$A\left(\%\right)=\frac{W_{wet}-W_{dry}}{W_{wet}}\times 100$$

##### Fourier Transform Infrared (FTIR)

To investigate the chemical bond change of the membrane, which occurred during the ion exchange and recast process, the FTIR analyses were conducted in the range of 500–4000 cm^−1^.

##### Ion-Exchange Capacity (IEC)

The ion-exchange capacity was measured by the titration method. The 1 cm^2^ dry membrane was immersed in 10 mL 0.01 M NaCl for 24 h. After removing the membrane, the remaining solution was titrated by 0.01 M NaOH, and phenolphthalein was used as the indicator. The IEC was calculated using Equation (6)

(6) $$I=\frac{0.01\times Vol_{NaOH}}{W_{dry}}$$

where *I* is the ion-exchange capacity (meq/g); *W_dry_* is the dry sample weight; and *Vol_NaOH_* is the titrant volume at endpoint (mL).

##### Ion Conductivity

Electrochemical impedance spectroscopy was performed to measure the ion conductivity. The membrane was held between two electrode probes connected to an AC power generator with a contacting area of 1.767 cm^2^. The Nyquist plot was recorded between 0.01–10^6^ Hz, and the amplitude was 10 mV. The resistance of the membrane was obtained from the plot. The proton conductivities of the membranes were calculated using Equation (7).

(7) $$\sigma =\frac{L}{R\times A}$$

where the σ is proton conductivity (Scm^−1^); *L* and *A* are the thickness (cm) and contacting area of the membrane (cm^2^), respectively; and *R* is the impedance of membrane (Ω), and this was obtained through the Precision Impedance Analyzer.

## 3. Results and Discussion

### 3.1. Circulation of Vanadium

#### 3.1.1. Ion Exchange Batch Equipment

**Figure 3:**
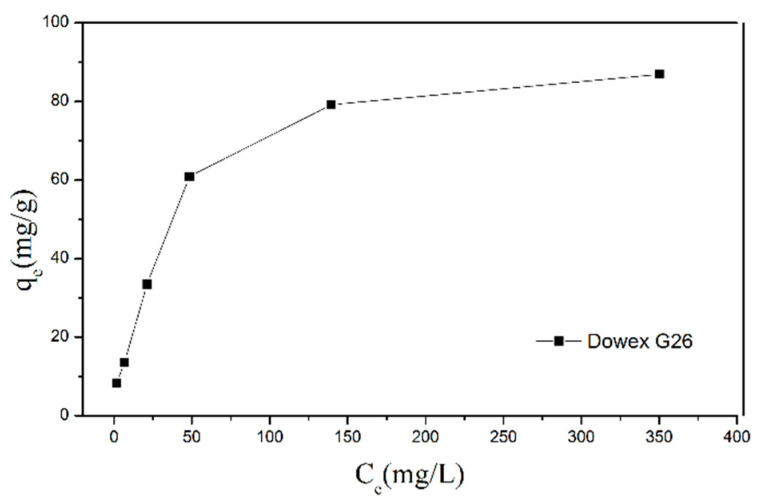
Isothermal adsorption curve of VO^2+^ through Dowex G26 resin.

In this study, 0.1 g resin was added to the six solutions with different initial concentrations of VO^2+^ (10, 20, 50, 100, 200, and 400 ppm) and adsorbed VO^2+^ for 24 h. The relationship between the *C_e_* (concentration of adsorbate in the liquid when adsorption is in equilibrium) and q_e_ (equilibrium adsorption capacity of the adsorbent) was used to create an isothermal adsorption curve (Figure 3). The result demonstrates that the maximum adsorption capacity was 86.9 mg/g.

**Figure 4:**
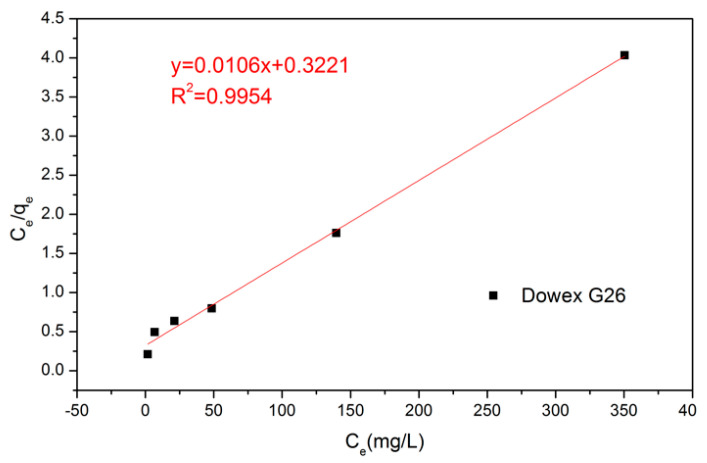
Langmuir isothermal model of VO^2+^ through Dowex G26 resin.

**Figure 5:**
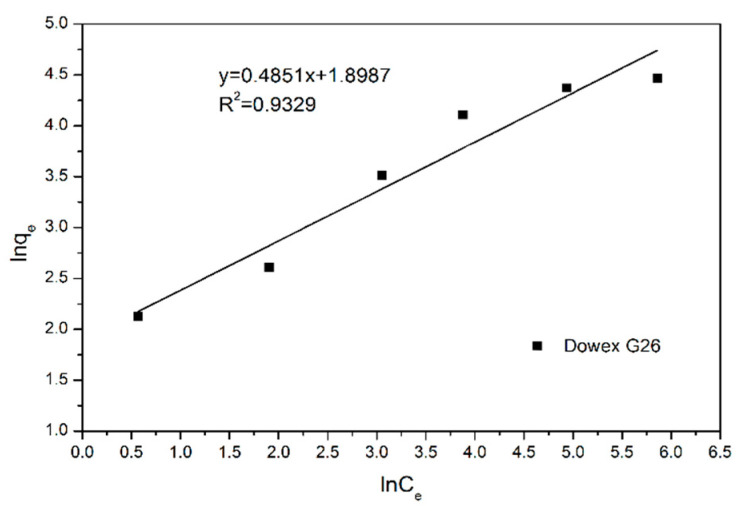
Freundlich isothermal model of VO^2+^ through Dowex G26 resin.

To obtain high accuracy of the maximum adsorption capacity and adsorptive behavior, Langmuir and Freundlich equations were used to create the figures [25,26]. Equation (8) and Figure 4 illustrate the Langmuir equation and linear regression between *C_e_* and *C_e_/q_e_*. The maximum adsorption capacity *q_m_* and adsorption equilibrium constant *K_L_* were gained by calculating with Equation (9) and linear regression equation (Table 4). Equation (9) and Figure 5 reveal the Freundlich equations and linear regression between *lnC_e_* and *lnq_e_*, and the empirical constant *n* and the adsorption equilibrium constant *K_F_* could be obtained (Table 4). According to the correlation coefficient R^2^ of two equations, the adsorptive behavior of Dowex G26 fits with the Langmuir model. It presents that the resin had a uniform adsorption position on the surface and that the theoretical maximum adsorption capacity was 94.34 mg/g.

(8) $$\frac{C_{e}}{q_{e}}=\frac{C_{e}}{q_{m}}+\frac{1}{q_{m}K_{L}}$$

(9) $$lnq_{e}=lnK_{F}+\frac{1}{n}lnC_{e}$$

At the eluted step, different molarities of HCl (0.1, 0.5, 1, 1.5, and 2) were used to desorb vanadium from the saturated resin. Table 5 demonstrates that the elution efficiency reached 99.5% by choosing 1 M HCl to desorb vanadium. The elution efficiencies decreased when using higher concentrations of acid because the strong acid may damage the resin. Under this condition, the optimal parameter of eluent molarity was chosen as 1 M HCl.

#### 3.1.2. Ion Exchange Column Equipment

**Figure 6:**
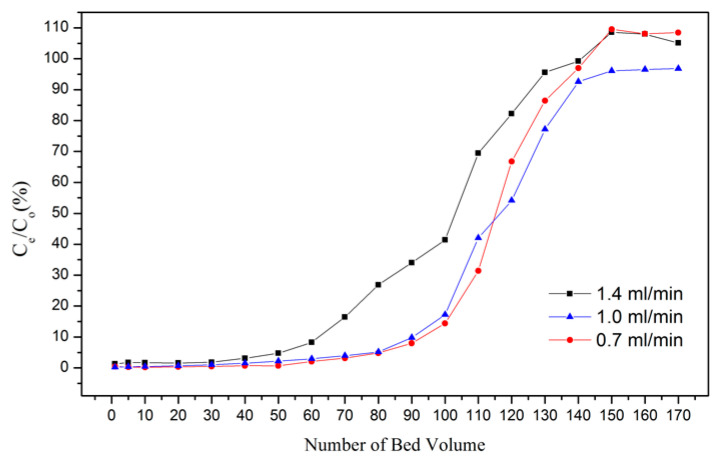
Adsorption breakthrough curve of VO^2+^ through Dowex G26 resin.

The column experiment was conducted under the optimal conditions in which Dowex G26 and 1 M HCl were used as the resin and eluent, separately, at room temperature. The results of the flow rate are illustrated in Figure 6. The breakthrough curves at flow rates of 0.7 and 1.0 mL/min demonstrated similar results in which the breakpoint (5% *C_e_*) was at 70 BV; however, the breakpoint at a flow rate of 1.4 mL/min was at 100 BV. The result represents that the increase in flow rate reduced the contact time between the resin surface and solution and caused a decrease in the adsorption capacity of the resins. Setting the flow rate at 1.0 mL/min had the optimal adsorption performance and economic benefit. Under this circumstance, the adsorption capacity was 81.86 mg/g.

**Figure 7:**
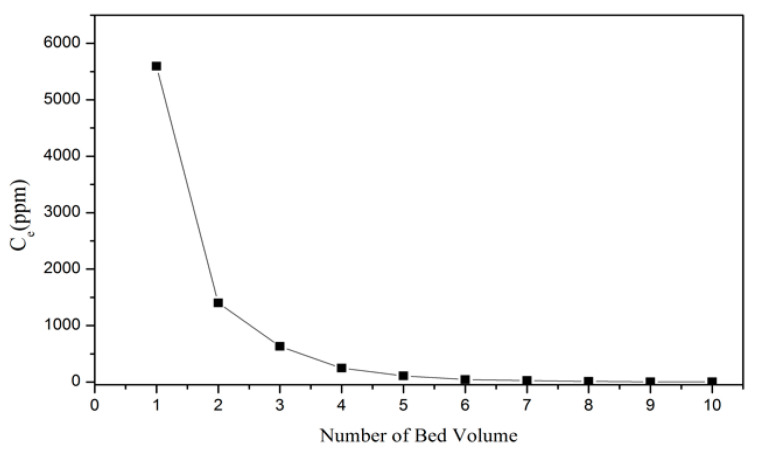
Eluting breakthrough curve of VO^2+^ through 1 M HCl.

In the column eluting experiment, 1 M HCl was used to elute vanadium from resin, and the eluting solution was then obtained after the process. The result of the eluting breakthrough curve is illustrated in Figure 7. The total BV was 5 BV, and the concentration of vanadium was 1596.72 ppm in the eluting solution. The eluting efficiency in this procedure was 97.5%, and the concentration ratio was 21.29. After the eluting process, H^+^ from HCl was adsorbed by the resin, and VO^2+^ was desorbed to the HCl solution. Through this procedure, the Dowex G26 resin could be regenerated and reused.

#### 3.1.3. Precipitation and Calcination

**Figure 8:**
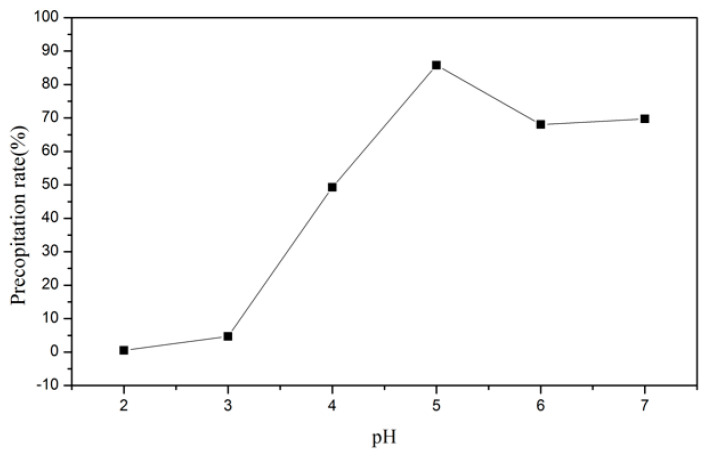
Precipitation rate of NH_4_VO_3_ with the pH value.

**Figure 9:**
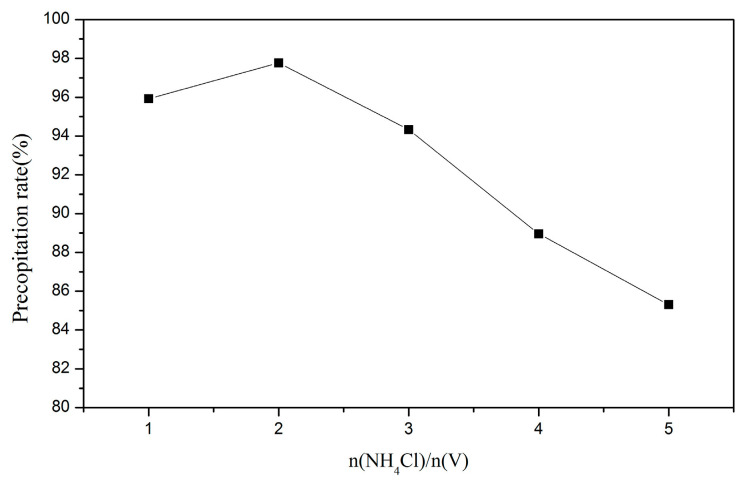
Precipitation rate of NH_4_VO_3_ with the amount of NH_4_Cl.

In this part, ammonium chloride was added to precipitate the NH_4_VO_3_. The pH value was adjusted by NH_4_OH and HCl, and the amount of ammonium chloride was controlled to receive the best precipitation rate. The results are demonstrated in Figure 8 and Figure 9. As a result, the optimal parameters were set as pH 5 and nNH_4_Cl:nV = 2:1. Under these conditions, the precipitation rate was 97.8%.

**Figure 10:**
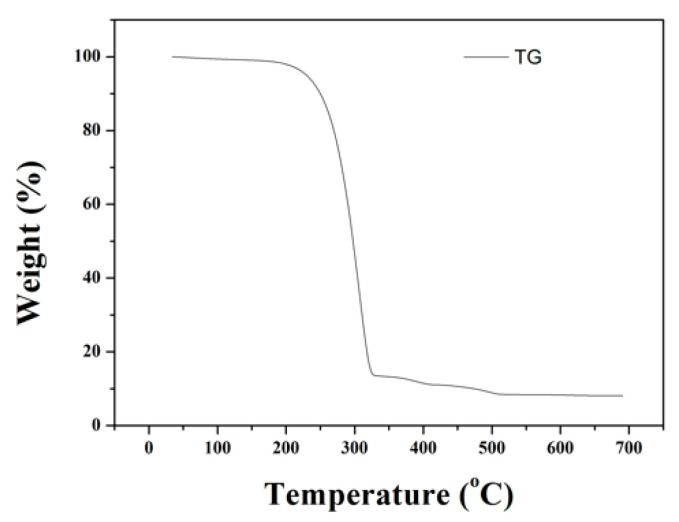
TG analysis of NH_4_VO_3._

**Figure 11:**
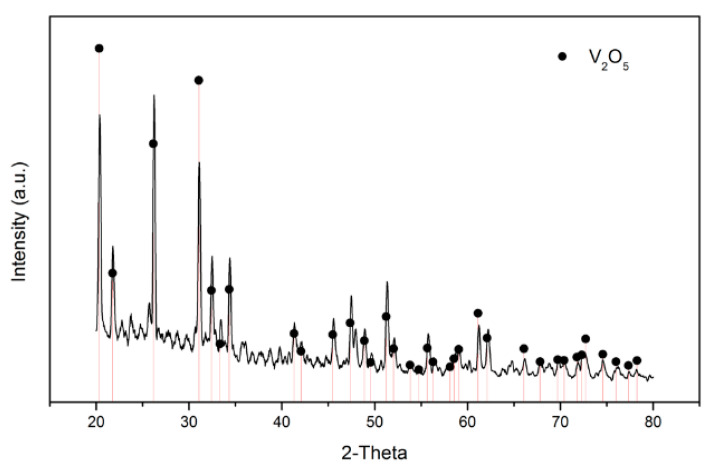
XRD analysis of V_2_O_5._

The NH_4_VO_3_ was achieved after precipitation, and the TG analysis was used to detect the calcination temperature. Based on the TG diagram (Figure 10), the temperature was set at 350 °C to make NH_4_VO_3_ ultimately become vanadium oxide (V_2_O_5_), and the V_2_O_5_ product could then be applied in many different areas [27,28]. The XRD analysis and purity of V_2_O_5_ are shown in Figure 11 and Table 6. The purity was higher than 99%, and the total recovery rate was 95.04%.

### 3.2. Recast Membrane Characterization

The vanadium-free membrane dispersion was recast into the solid recycled membrane. The various characteristic analyses were done to verify the reusability of the recycled membrane. The same analyses were conducted on the commercial membrane and waste membrane for comparison. The results are shown below.

#### 3.2.1. Membrane Moisture

Membrane moisture is one of the critical parameters affecting the proton conduction and mechanical stability of the membrane. The moisture of different membranes is presented in Table 7. As shown in Table 7, the moisture of the recycled membrane was familiar to the commercial membrane. The waste membrane has higher moisture than the recycled membrane because the vanadium ion fouling in the waste membrane could be formed with water molecules. Therefore, the vanadium penetration would not lead to a moisture decrease.

#### 3.2.2. FTIR Spectra

**Figure 12:**
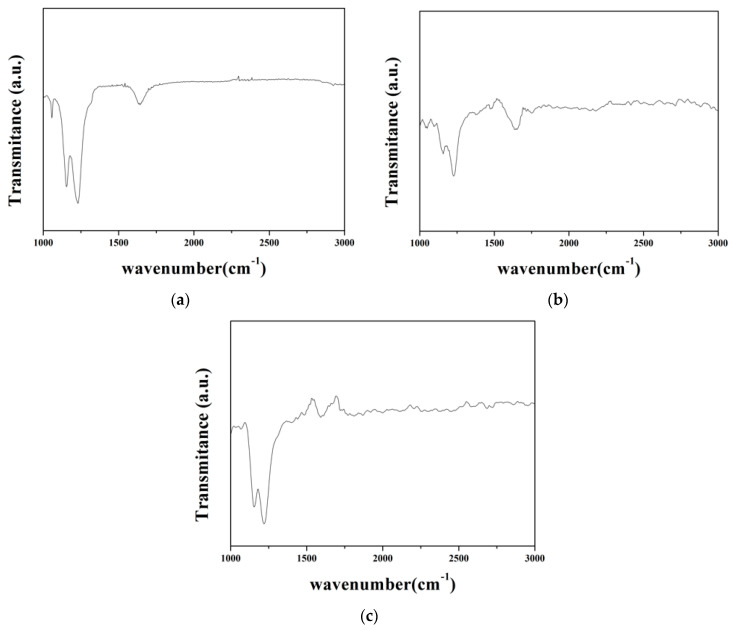
FTIR spectra in the range of wavenumbers 1000–3000 cm^−1^ of (**a**) commercial membrane, (**b**) recycled membrane, and (**c**) waste membrane.

**Figure 13:**
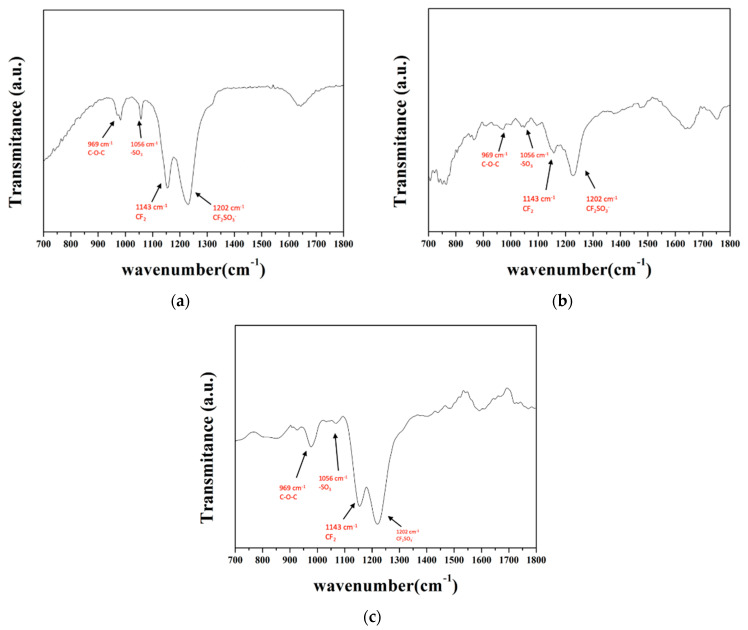
FTIR spectra in the range of wavenumbers 700–1800 cm^−1^ of (**a**) commercial membrane, (**b**) recycled membrane, and (**c**) waste membrane.

FTIR was used in this study to analyze the chemical bonding of the membrane to verify if the microstructure of the recycled membrane was affected. The results are presented in Figure 12 and Figure 13, in which Figure 12a–c shows the spectra in the range of wavenumbers 1000–3000 cm^−1^_,_ and Figure 13a–c illustrates the identical spectra but focuses on the range of wavenumbers 700–1800 cm^−1^.

The absorption peaks and corresponding bonds of Nafion spectra are listed in Table 8. As shown in Figure 12, the prominent characteristic peaks of the commercial membrane meet the Nafion spectra. The characteristic peaks of the recycled membrane matched Nafion spectra as well; however, the absorption intensity appeared weaker. The reason may be caused by the microstructure change during the ion-exchange process and membrane recast. The absorption spectra of the waste membrane were familiar to the commercial membrane; however, the characteristic peak at wavenumber 1056 cm^−1^ was not evident in Figure 13 because the vanadium occupied the sulfonic acid groups.

#### 3.2.3. Ion-Exchange Capacity

The ion-exchange capacity (IEC) was used to evaluate the ion exchangeability of sulfonic acid groups in the proton-exchange membrane. The IEC may be affected by the total amount of sulfonic acid groups and the degree of exposure. Typically, the higher IEC represented the higher ion conductivity. The results are shown in Table 9, and the IEC of the waste membrane was only 80% of the commercial membrane, which means the acid groups were occupied by vanadium ion; therefore, the ion exchange could not be performed. The IEC of the recycled membrane reached 90% of the commercial membrane, which means the IEC increased because the acid groups were released after adsorbing vanadium ions through the ion-exchange process.

#### 3.2.4. Ion Conductivity

The ion conductivity was one of the indicators to evaluate whether the membrane could be used as the proton-exchange membrane of the vanadium flow battery. The ion conductivity was calculated by Equation (7) after measuring the impendence of the membrane. The impendence was obtained by electrochemical impedance spectroscopy.

**Figure 14:**
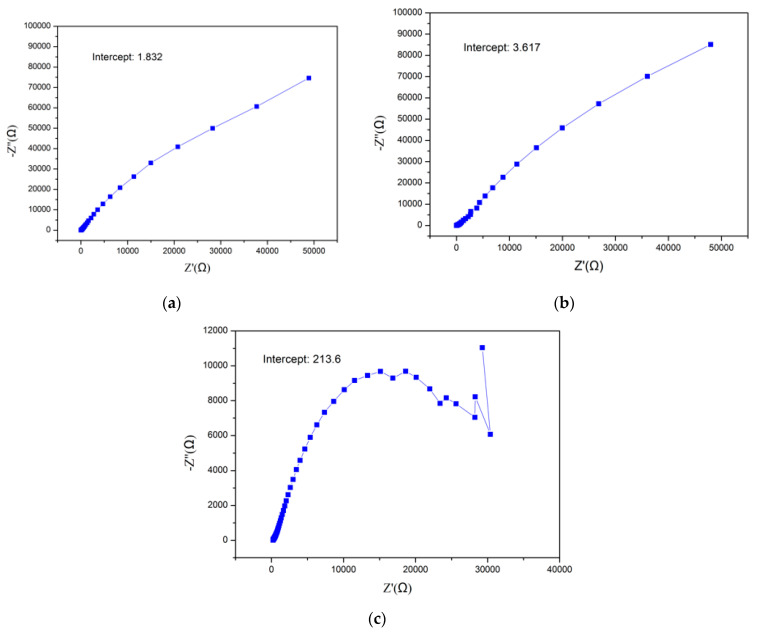
The Nyquist of (**a**) a commercial membrane, (**b**) recycled membrane, and (**c**) waste membrane.

**Figure 15:**
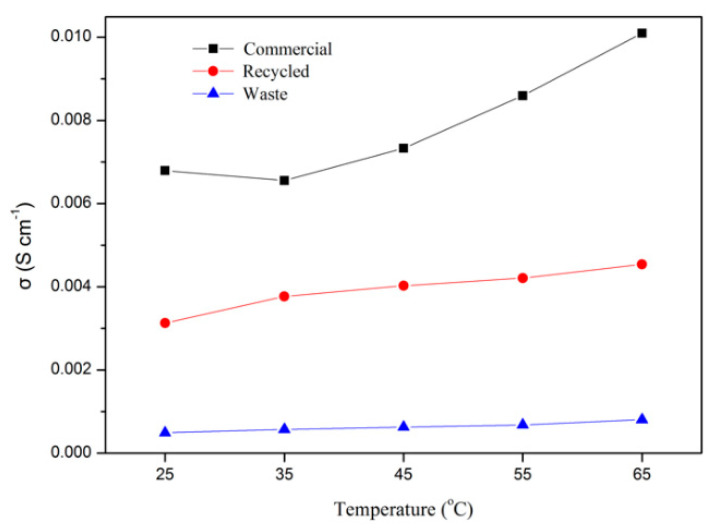
The comparison of ion conductivity of three membranes at different temperatures.

The Nyquist plots of three membranes are presented in Figure 14, and the results of the ion conductivity are presented in Figure 15. As shown in Figure 15, the ion conductivity of the recycled membrane was half of the commercial membrane, which was 6.6-times larger than the waste membrane. The ion conductivity of the recycled membrane increased because the vanadium ion was removed through Dowex G26 resin; however, it could not reach the same level as the commercial membrane. This behavior could be explained as the microstructure change occurring during the ion exchange or recast process, decreasing the ability of proton transport in the membrane.

## 4. Conclusions

This study is divided into two parts: the recovery of vanadium by ion exchange and the recast of the proton-exchange membrane.

1. In the ion-exchange batch experiment, according to the results of the adsorption isotherms, the adsorptive behavior of Dowex G26 fit with the Langmuir model. This indicates that the resin had a uniform adsorption position on the surface and that the theoretical maximum adsorption capacity was 94.34 mg/g.
2. In the ion-exchange column experiment, the optimal parameters were set at the flow rate of 1 mL/min, in which the adsorption capacity was 81.86 mg/g. During the eluting process, 1 M HCl was chosen as the eluent, and the eluting efficiency was 97.5%. The concentration of the enrichment solution was 1596.72 ppm.
3. In the precipitation process, the optimal parameters were set as pH = 5 and NH_4_Cl:V = 2:1. In this case, the precipitation rate was 97.8%. The calcination temperature was set at 350 °C, and the purity of the V_2_O_5_ was over 99%.
4. The recycled membrane was obtained by recasting the vanadium-free membrane dispersion, and the characteristics of the membranes were studied. The membrane moisture of the recycled membrane was 25.98%, and the IEC was 0.565 meq/g. The results of FTIR and ion conductivity revealed that the vanadium in the recycled membrane was eliminated by the ion-exchange method; however, the microstructure of the recycled membrane was influenced during ion exchange or recasting.

By using the ion-exchange method, vanadium could be removed efficiently, and high purity of the vanadium product was obtained after enrichment, precipitation, and calcination, and this could be reused as a raw material in industry. The ion-exchange capacity and ion conductivity of the recycled membrane were significantly ameliorated; however, there is still room for improvement to reach the same level as the commercial membrane. In summary, the vanadium and membrane were recovered simultaneously from the waste vanadium flow battery. This research has great potential toward the goal of waste reduction and resource circulation.

## Figures and Tables

**Table 1:** The information of recovering the proton-exchange membrane from fuel cell batteries.

Author	Description
Xu F. et al. [13]	The catalyst-coated membrane was dipped into sulfuric acid until the formation of transparent solution composed of Pt and perfluorosulfonic acid resin. The membrane was dissolved, and the amorphous carbon nanoparticles as catalyst supports in catalyst layers were oxidized. Subsequently, both metal Pt and perfluorosulfonic acid resin were separated by centrifugal separation. Then, the resin was recast into a membrane, and the single fuel cell performance was tested.
Moghaddam J. A. et al. [14]	The three different dissolution methods were used to resolve the Nafion membranes, and then the obtained solutions were cast. The prepared different recast Nafion membranes were evaluated by water uptake measurement, swelling behaviors, ion-exchange capacity, and proton conductivity at different temperatures. The results showed the different recast Nafion membranes had more appropriate water uptake, proton conductivity, and chemical and mechanical stability levels compared with the commercial Nafion membrane.
Silva R. et al. [15]	Perfluorosulfonate ionomer dispersions in three different solvents (ethylene glycol, dimethyl sulfoxide, and dimethylformamide) were used to prepare solution-cast membranes. The dispersions were obtained by dissolution of Nafion^®^ 112 membranes in a reactor using a water–ethanol solution. The results show that all cast samples had lower chemical stability compared with commercial membranes. Moreover, only membranes cast from dimethylformamide-based dispersions gave conductivity performance comparable to those of Nafion^®^ 112 and 115.
Laporta M. et al. [16]	In the present study, some procedures for preparing a Nafion water dispersion, starting from a Nafion-117 membrane, are described. The morphological characteristics of the prepared dispersions were compared with Nafion commercial dispersion (NCD). Moreover, membranes with a thickness of 5–20 μm were prepared and characterized, using both the obtained and the NCD dispersions. The obtained data showed that Nafion water dispersion, which can be used to prepare the membrane/electrode system, resulted in thin membranes that absorb more water than NCD membranes and have equal and/or higher proton conduction compared with the NCD.

**Table 2:** Different techniques of recovering vanadium through resins.

Author	Resin	Description
Zeng L. et al. [17]	D314	The loading of V on weak base resin D314 from sulfuric acid leach solutions of stone coal containing 2.06 g/L V (V_2_O_5_) was found to be 260 mg/mL with a contact time of 60 min at pH = 4, giving a recovery of 99%.
Li W. et al. [18]	ZGA414 D202 D453 D301FC ZGA351	Anion-exchange resin ZGA414 was tested as its optimum adsorption capacity compared with D202, D453, D301FC, and ZGA351 resins. Ion exchange tests indicated that only V(V) was loaded from the synthetic solution at pH > 1.5, while it was difficult to separate V(V) from Fe(III), which also made the resin toxic.
Fan Y. et al. [19]	D314	To recover vanadium from vanadium-containing chromate solution, the separation of vanadium from chromium using the weak base resin D314 both in batch and column test was studied. Experimental results showed that, in the pH range of 2.5–6.5, by double-adsorption with the resin, vanadium and chromium can be completely separated and recovered from vanadium-containing chromate solution.
Fritz J. S. et al. [20]	Dowex 50W-x8	Vanadium is quantitatively removed as a vanadium(V)^−^ hydrogen peroxide complex; the other metal ions are eluted later with stronger acids. Varying ratios of vanadium(V) to iron(lll) up to 1:100 are separated.
Drużyński S. et al. [21]	Dowex 1-x8	Three types of polymer strongly acidic ion exchangers were used. The ion-exchange resins differed in terms of granularity and their ion-exchange capacity. As a result, breakthrough curves were made for three main components of the test extract, i.e., ions of vanadium, iron, and potassium. On this basis, the optimum conditions for the removal of iron ions from the solution were defined, and the technological concept of the process in the semitechnical scale was proposed.

**Table 3:** Characterization information of the waste Nafion 117 membrane dispersion.

Characterization	Data
Concentration of membrane	2.5%
Concentration of vanadium	74 mg/L
pH value	2.01
ORP value	300 mV

**Table 4:** The data of the Langmuir model and Freundlich model.

Langmuir Model (R^2^ = 0.9954)	Freundlich Model (R^2^ = 0.9329)
*q_m_* = 1/slope 1/0.0106 = 94.34 mg	*n* = 1/slope 1/0.4851 = 2.06
*K_L_* = 1/(*q_m_* × intercept) 1/(94.34 × 0.3221) = 0.0329	*K_F_* = *e*^intercept^ *e*^1.8987^ = 6.68

**Table 5:** Elution efficiencies of different molarities of HCl.

Eluent Molarity of HCl (M)	Elution Efficiency (%)
0.1	76.9
0.5	98.5
1	99.5
1.5	95.3
2	79.3

**Table 6:** Composition of V_2_O_5._

Compounds	Content (%)
V_2_O_5_	99.09
Na_2_O	0.41
MgO	0.19
K_2_O	0.31
Fe_2_O_3_	<0.01

**Table 7:** The moisture of different membranes.

	*W_dry_*	*W_wet_*	Moisture (%)
Recycled membrane	0.0364	0.051	25.98
Commercial membrane	0.0384	0.054	28.89
Waste membrane	0.0341	0.043	26.55

**Table 8:** The absorptions peaks and corresponding bonds of the Nafion spectra.

Wavenumber (cm^−1^)	Chemical Bonds
969	C-O-C
1056	-SO_3_H
1143	CF_2_
1202	CF_2_SO_3_

**Table 9:** Ion-exchange capacity of the three membranes.

	Weight (g)	IEC (meq/g)
Recycled membrane	0.0248	0.565
Commercial membrane	0.0231	0.628
Waste membrane	0.0256	0.508

## Data Availability

Not applicable.
